# Low-Molecular-Weight Peptides Prepared from *Hypsizygus marmoreus* Exhibit Strong Antioxidant and Antibacterial Activities

**DOI:** 10.3390/molecules29143393

**Published:** 2024-07-19

**Authors:** Shaoxiong Zhou, Zheng Xiao, Junzheng Sun, Longxiang Li, Yingying Wei, Mengjie Yang, Yanrong Yang, Junchen Chen, Pufu Lai

**Affiliations:** 1 College of Food Science, Fujian Agriculture and Forestry University, Fuzhou 350002, China; zhousx2023@163.com (S.Z.); wyy787459024@163.com (Y.W.); yummy2023yummy@163.com (M.Y.); 2 Institute of Food Science and Technology, Fujian Academy of Agricultural Sciences, Fuzhou 350003, China; ethyxwat@163.com (Z.X.); sunjzll@163.com (J.S.); 13616901013@163.com (L.L.); 18960973035@163.com (Y.Y.); junchenccc@163.com (J.C.); 3 National R & D Center for Edible Fungi Processing, Fuzhou 350003, China; 4 Key Laboratory of Subtropical Characteristic Fruits, Vegetables and Edible Fungi Processing (Co-Construction by Ministry and Province), Ministry of Agriculture and Rural Affairs, Fuzhou 350003, China

**Keywords:** *Hypsizygus marmoreus*, peptides, antioxidant, antibacterial

## Abstract

*Hypsizygus marmoreus* has abundant proteins and is a potential source for the development of bioactive peptides. However, currently, the research on the bioactive components of *H. marmoreus* mainly focuses on polysaccharides, and there is no relevant research on the preparation of bioactive peptides. In this article, an ultrasound-assisted extraction method was used to extract proteins from *H. marmoreus*, and then, four peptides with different molecular weight ranges were prepared through protease hydrolysis and molecular classification. The antioxidant and antibacterial activities were also studied. Under the optimal conditions, the extraction rate of *H. marmoreus* proteins was 53.6%. Trypsin exhibited the highest hydrolysis rate of *H. marmoreus* proteins. The optimal parameters for enzymatic hydrolysis were a substrate concentration of 3.7%, enzyme addition of 5700 U/g, pH value of 7, extraction temperature of 55 °C, and time of 3.3 h. Under these conditions, the peptide yield was 59.7%. The four types of *H. marmoreus* peptides were prepared by molecular weight grading. Among them, peptides with low molecular weight (<1 kDa) had stronger antioxidant and antibacterial activities. This study provides a theoretical basis for the efficient preparation of *H. marmoreus* peptides and the development of antioxidant and antibacterial peptide products.

## 1. Introduction

*Hypsizygus marmoreus*, also known as seafood mushroom or crab-flavored mushroom, is a large saprophytic fungus belonging to the phylum of Basidiomycota and the genus of Agaricus [1,2,3]. It has a crispy and tender texture, a delicious taste, and rich nutrients, and it is considered a kind of healthy food widely loved by customers [4,5]. *H. marmoreus* is rich in various nutrients, such as protein, polysaccharides, fat, dietary fiber, vitamins, minerals, flavonoids, etc., and plays a good role in antioxidant, anti-cancer, antihyperlipidemia, and other functions [6,7,8,9,10,11,12].

After incomplete degradation of proteins, peptides with varying lengths are obtained, some of which are composed of 2–20 amino acid residues and often have multiple biological functions, known as bioactive peptides [13,14,15]. The type and intensity of their biological activity are influenced by different characteristics, such as amino acid composition, sequence, charge, hydrophobicity, etc. [16,17]. Bioactive peptides can exert various biologically beneficial effects [18], such as anti-tumor [19], antioxidant [20], antiosteoporosis [21], antibacterial [22], antithrombotic, immunomodulatory, etc. [23]. Therefore, edible mushroom peptides have great application prospects in nutritional functional foods [24], medical medicine [25], and other fields. Bioactive peptides can protect consumer health through the regulation or enhancement of human physiological functions [26]. The functions of bioactive peptides from edible mushrooms have been reported. Peptides produced via enzymatic hydrolysis of *Ganoderma lucidum* proteins have antioxidant properties [27]. Peptides extracted and purified from the mycelium of *Pleurotus eryngii* exhibit anti-tumor, antioxidant, and immunostimulatory activities [28]. The crude protein content of *H. marmoreus* accounts for 27.3% [29], which provides powerful conditions for the preparation of peptides and is a potential source for the development of bioactive peptides. However, currently, the research on the bioactive components of *H. marmoreus* is mostly focused on polysaccharides [30,31,32], phenolic substances [33], and indole compounds [34], and there are few reports on the study of bioactive peptides from *H. marmoreus*.

In the present work, *H. marmoreus* was used as raw material to extract proteins, and then, peptides were prepared through enzymatic hydrolysis. The parameters of enzymatic hydrolysis were optimized through single-factor and response surface experiments to obtain the optimal process for peptide preparation. The antioxidant and antibacterial activities of peptides with different molecular weights were analyzed in detail, providing a reference for the development of bioactive peptides from *H. marmoreus*.

## 2. Results

### 2.1. Extraction Results of H. marmoreus Protein

An L_16_(4^5^) orthogonal table was selected based on single-factor experiments to optimize the extraction conditions using protein extraction rate as the indicator. The result analysis is shown in Table 1 and Table 2.

According to the extreme difference in Table 1, all four selected factors had an impact on the extraction rate, in the order of D > A > C > B, which was ultrasonic time > solid–liquid ratio > ultrasonic power > pH value. According to the analysis of variance in Table 2, the effects of ultrasonic time and solid–liquid ratio on the extraction rate of protein reached a highly significant level, while the effects of pH value and ultrasonic power were not significantly different. At the same time, it was known that the optimal process combination for extracting protein from *H. marmoreus* was A_3_B_4_C_3_D_3_, which included a solid–liquid ratio of 1:35, pH value of 13, ultrasonic power of 300 W, and ultrasonic time of 35 min.

To verify the reliability of the optimal combination, three experiments were conducted under the optimal combination conditions, and the average protein extraction rate was 53.6%, which was higher than all experimental designs in Table 1, indicating that the optimization results were correct.

### 2.2. Preparation of H. marmoreus Peptides

#### 2.2.1. Screening of Proteases

**Figure 1:**
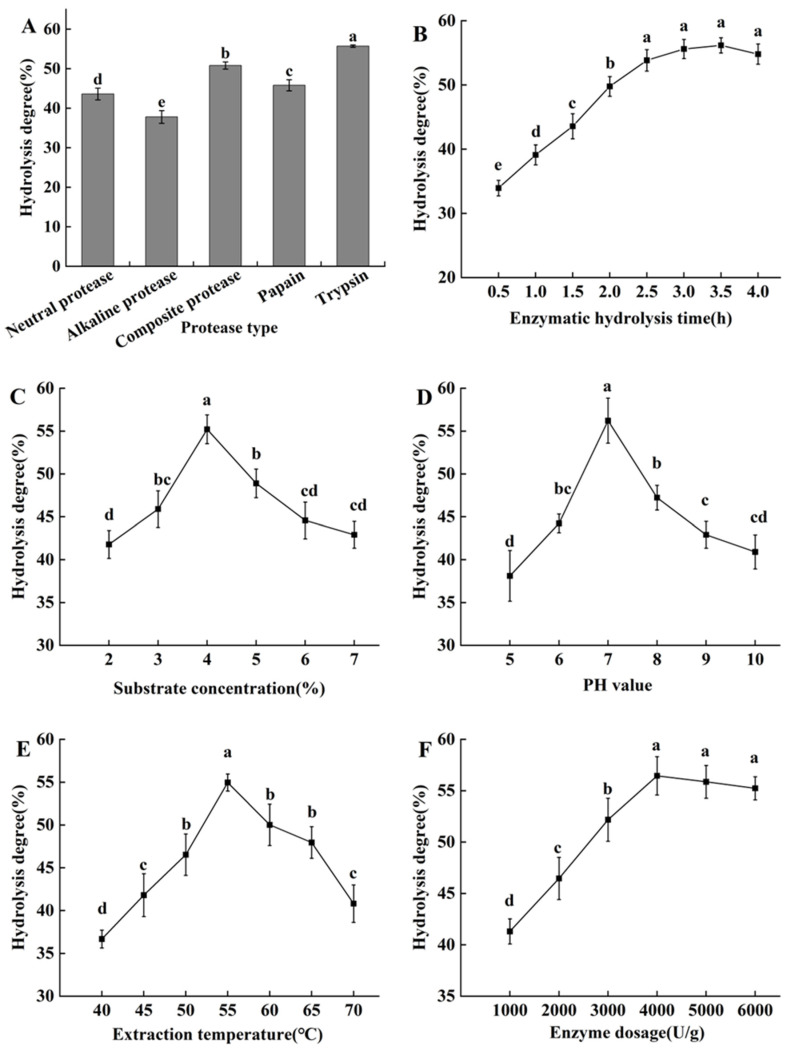
The optimization of parameters of *H. marmoreus* protein hydrolysis: (**A**) Proteases, (**B**) enzymatic hydrolysis time, (**C**) substrate concentration, (**D**) pH value, (**E**) extraction temperature, and (**F**) enzyme dosage. Different letters in the same indicators indicate significant differences (*p* < 0.05).

Due to the different cleavage sites and characteristics of different enzymes, proteins can be decomposed in different ways, resulting in different enzymatic hydrolysis effects. The hydrolysis degree of *H. marmoreus* protein by different proteases under their respective optimal conditions is shown in Figure 1A. Trypsin and composite protease showed good protein degradation ability, followed by papain and neutral protein, while alkaline protease was relatively weak. The results showed that the hydrolysis degree of *H. marmoreus* protein by trypsin was 55.2%, significantly higher than the other four enzymes; therefore, it was selected for subsequent experiments.

#### 2.2.2. The Effects of Enzymatic Hydrolysis Duration Time

With the extension of enzymatic hydrolysis time, the *H. marmoreus* protein was hydrolyzed under the catalytic action of enzymes, increasing the hydrolysis degree (Figure 1B). The degree of hydrolysis decreased after 3.5 h, but it was not significant (*p* > 0.05). In the beginning, the contact and reaction between the enzyme and substrate were insufficient, resulting in a low hydrolysis degree. As the enzymatic hydrolysis time was prolonged, the enzyme came into full contact with the substrate, and more and more amino acid sites were cleaved, increasing the hydrolysis degree. But when the time was longer than 3 h, the enzymatic hydrolysis was excessive, and the peptides were broken down into amino acids. Since there was no significant difference in hydrolysis degree between 3 h, 3.5 h, and 4 h (*p* > 0.05), from the perspective of energy conservation and time saving, 3 h was considered as the optimal enzymatic hydrolysis duration time.

#### 2.2.3. The Effects of Substrate Concentration

The concentration of substrates can also affect the rate of enzymatic reactions. The higher the substrate concentration, the faster the enzymatic reaction rate until it reaches saturation. The *H. marmoreus* protein hydrolysis rate reached its maximum value of 55.2% under the condition of substrate concentration of 4% (Figure 1C). When the substrate concentration was less than 4%, as the substrate concentration increased, more and more substrates were catalyzed and degraded by enzymes, increasing hydrolysis products. When the substrate concentration exceeded 4%, there were not enough enzymes to catalyze substrate hydrolysis, resulting in insufficient hydrolysis and a decrease in hydrolysis degree. Therefore, the substrate concentration of 4% was appropriate.

#### 2.2.4. The Effect of Different pH

The pH value of the reaction system is an important factor affecting enzyme activity. Enzymes undergo different dissociation states in different pH reaction systems, which directly affect the catalytic effects. If the pH value of the reaction system is not within the optimal range of the enzyme, the conformation of enzyme molecules may change, which can inhibit enzyme activity and lead to a decrease in hydrolysis degree. When the pH was between 5 and 7, the degree of *H. marmoreus* protein hydrolysis increased with pH (Figure 1D). When the pH was 7, the degree of hydrolysis reached its maximum value of 56.2%. But when the pH value was greater than 7, the biological activity of the enzyme decreased, and its hydrolysis ability also decreased. The pH value of 7 was chosen for subsequent experiments.

#### 2.2.5. The Effect of Extraction Temperature

The maximum *H. marmoreus* protein hydrolysis degree of 54.9% was obtained at the temperature of 55 °C (Figure 1E). Due to the temperature dependence of enzymes, an increase in temperature below the optimal temperature increased the enzyme activity and the number of collisions between enzyme molecules and substrates, thereby increasing the rate of enzymatic reactions. Generally, enzymes belong to proteins. When the temperature was too high, the spatial structure of the enzyme was disrupted, leading to enzyme denaturation and inactivation, resulting in a decrease in hydrolysis degree. Therefore, it was better to choose an extraction temperature of 55 °C.

#### 2.2.6. The Effect of Enzyme Dosages

As shown in Figure 1F, with the increase in enzyme dosage, the substrate hydrolysis degree showed an initial increase, followed by a gradual and decreasing trend. The optimal enzyme dosage was 4000 U/g. Beyond 4000 U/g, the increase became insignificant (*p* > 0.05). In the reaction system, when the enzyme concentration was relatively low, as the amount of enzyme increased, the binding sites between the enzyme and substrate continued to increase, and the rate of enzyme reaction gradually increased, leading to more and more proteins being hydrolyzed into peptides. When the amount of enzyme exceeded a certain value, due to the constant substrate concentration, the binding sites tended to saturate. At this time, even if the enzyme amount continued to increase, the degree of hydrolysis would not be significantly affected.

#### 2.2.7. Response Surface Analysis Scheme and Regression Model

The response surface analysis test plan and results are shown in Table 3. According to multiple regression analysis, the impact of each experimental factor on the response value was not a simple linear relationship. After regression fitting, the multiple quadratic regression equation between each factor and the comprehensive value was obtained:

Y = 56.74 + 2.34X_1_ − 4.72X_2_ + 0.5794X_3_−0.2212X_4_ + 3.62X_5_ − 0.77X_1_X_2_ + 0.2175X_1_X_3_ + 0.0225X_1_X_4_ + 1.68X_1_X_5_ + 0.185X_2_X_3_ − 0.0275X_2_X_4_ − 0.66X_2_X_5_ + 0.19X_3_X_4_ − 0.88X_3_X_5_ + 0.165X_4_X_5_ − 2.76X_1_^2^ − 9.51X_2_^2^ − 4.65X_3_^2^ − 4.10X_4_^2^ − 3.43X_5_^2^

According to Table 4, the response value Y (degree of hydrolysis) model was highly significant. In the first degree, X_1_ (enzyme hydrolysis time), X_2_ (substrate concentration), and X_5_ (enzyme dosage) reached extremely significant levels (*p* < 0.01). X_3_ (pH value) and X_4_ (extraction temperature) were not significant. X_1_^2^, X_2_^2^, X_3_^2^, X_4_^2^, and X_5_^2^ all reached extremely significant levels (*p* < 0.01). Among the interaction terms, only X_1_X_5_ reached a significant level (*p* < 0.05), while the other interaction items were not significant. Therefore, subsequent analysis will only focus on the interaction between X_1_ and X_5_. The order of influence of five factors on the comprehensive value was X_2_ (substrate concentration) > X_5_ (enzyme dosage) > X_1_ (enzymatic hydrolysis time) > X_3_ (pH value) > X_4_ (enzymatic hydrolysis temperature). The F-test and loss-of-fit test results of the quadratic regression equation revealed that a highly significant model (*p* < 0.01) and an insignificant loss-of-fit term (*p* > 0.05) indicated a good fit of the model. Therefore, the hydrolysis degree of seafood mushroom protein under different extraction conditions could be analyzed and predicted using this model.

**Figure 2:**
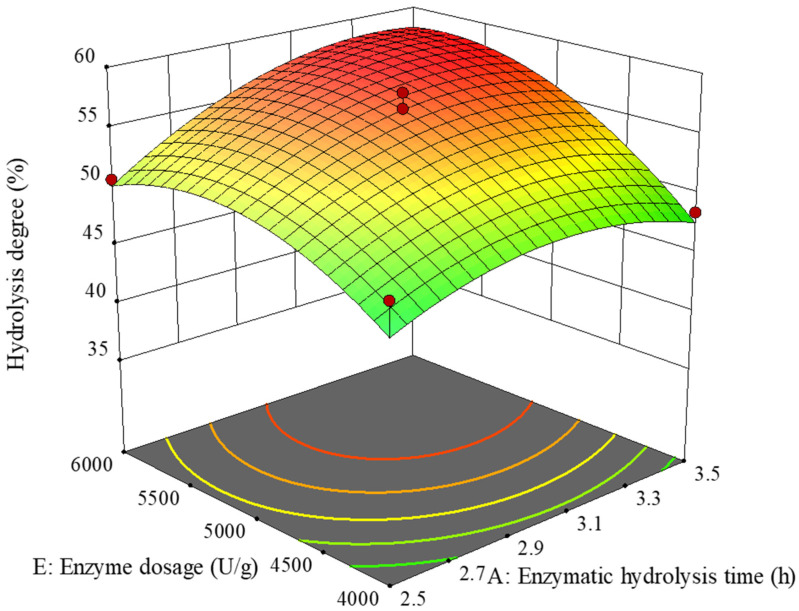
The effect of the interaction between hydrolysis time and enzyme dosage on hydrolysis degree.

Figure 2 shows the effect of the interaction between enzymatic hydrolysis time and enzyme dosage on the hydrolysis degree. The curves of enzymatic hydrolysis time and enzyme dosage were both steep, indicating that their effects on hydrolysis degree were significant. The change in hydrolysis degree with enzyme addition was greater than the hydrolysis time, indicating that the enzyme addition had a more significant impact on hydrolysis degree, which was consistent with the results of the above analysis of variance.

The optimal combination of factor levels could be predicted by regression equations. The optimal enzymatic hydrolysis conditions for seafood mushroom protein were obtained by converting the levels of each factor into corresponding measured values, which were a time of 3.34 h, substrate concentration of 3.7%, pH value of 7, temperature of 54.95 °C, and enzyme addition of 5725 U/g. Under these conditions, the hydrolysis degree was 59.6%. Considering the feasibility of practical operation, the optimal extraction process was revised to enzymatic hydrolysis time of 3.3 h, substrate concentration of 3.7%, pH value of 7, temperature of 55 °C, and enzyme addition of 5700 U/g. According to the revised optimal process conditions, six repeated validation experiments were conducted, and the hydrolysis degree was measured to be 59.7%. The absolute error between this value and the theoretical prediction value was less than 5%, and the difference in the *t*-test results was not significant, which further verified the reliability of the model.

### 2.3. In Vitro Antioxidant Test

#### 2.3.1. 1,1-Diphenyl-2-Picrylhydrazyl (DPPH) Free Radical Scavenging Ability

**Figure 3:**
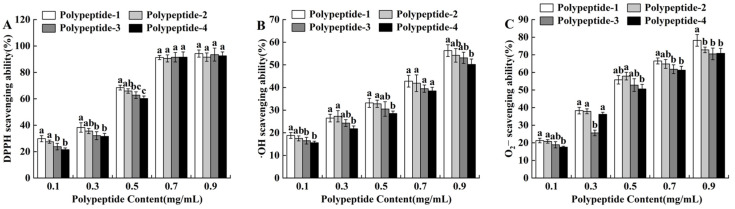
Scavenging rates *H. marmoreus* peptides on DPPH (**A**), •OH (**B**), ·O_2_^−^ (**C**) radicals. Different letters in the same indicators indicate significant differences (*p* < 0.05).

As shown in Figure 3A, the DPPH radical scavenging ability of seafood mushroom peptides with different molecular weights increased with concentration. Peptide solutions with high concentration had high free radical scavenging rates. When the peptide content was within 0.1–0.5 mg/mL, the DPPH free radical scavenging rate of peptide-1 was higher than the other three peptides. This was perhaps because the higher the proportion of hydrophobic amino acids in low relative molecular weight peptides, the stronger their ability to interact with polyunsaturated fatty acids, resulting in better scavenging effects of peptides on free radicals [35]. When the peptide concentration was 0.7 mg/mL, the scavenging ability of seafood mushroom peptides with different molecular weights on DPPH • free radicals reached over 90%. When the peptide content was greater than 0.7 mg/mL, the DPPH scavenging ability of four different molecular weight peptides was slightly enhanced, but the change was not significant (*p* > 0.05). This indicated that the molecular weight of seafood mushroom peptides had a high impact on DPPH scavenging ability.

#### 2.3.2. Determination of •OH Radical Scavenging Ability

As shown in Figure 3B, the •OH scavenging ability of seafood mushroom peptides increased with their concentration. Peptide solutions with high concentration had a higher •OH radical scavenging rate, but the •OH scavenging ability of peptides with different molecular weights was not significantly different at the same peptide concentration. Except for 0.3 mg/mL, the •OH scavenging ability order of peptides at other concentration levels was polypeptide-1 > polypeptide-2 > polypeptide-3 > polypeptide-4, which was consistent with the research results showing that *Hericium erinaceus* peptides with smaller molecular weights had a stronger antioxidant activity [36]. When the peptide concentration was 0.7 mg/mL, the •OH scavenging ability of peptides with different molecular weights was at a relatively low level and did not reach 50%.

#### 2.3.3. Determination of ·O_2_^−^ Radical Scavenging Ability

The ·O_2_^−^ scavenging ability of seafood mushroom peptides with different molecular weights also showed a dose–response relationship, namely, the higher the concentration of the peptide solution, the higher the ·O_2_^−^ scavenging rate (Figure 3C). When the peptide concentration was 0.3 mg/mL, the ability of polypeptide-3 to clear ·O_2_^−^ was significantly lower than the other three peptide solutions. When the peptide concentration was 0.9 mg/mL, the ·O_2_^−^ scavenging ability of peptides with different molecular weights was at a high level, reaching over 70%, which was equivalent to the ability of *Ganoderma lucidum* fruit peptides to scavenge superoxide free radicals [37]. Polypeptide-1 showed significant differences compared with polypeptide-2, polypeptide-3, and polypeptide-4. The results indicated that the molecular content of seafood mushroom peptides had a significant impact on the ·O_2_^−^ scavenging ability.

### 2.4. Antibacterial Activity of H. marmoreus Peptides

*H. marmoreus* peptides with different molecular weights exhibited different inhibitory activities against bacteria and fungi (Table 5). Peptide 1 had the strongest antibacterial activity, and its inhibitory activity against *Staphylococcus aureus*, *Salmonella typhimurium*, *Escherichia coli*, and *Pseudomonas aeruginosa* was significantly higher than other peptides (*p* < 0.05). Polypeptide-4 had the best inhibitory activity against *Aspergillus niger*, while peptide 3 had no inhibitory effect on *Aspergillus niger*. In addition, polypeptide-2 and polypeptide-4 had no inhibitory effects on *Escherichia coli* and *Staphylococcus aureus*, respectively. Overall, small molecule peptides had more significant antibacterial effects, which might be due to their easier proximity to the surface of micro-organisms or the formation of active molecular structures, thereby exerting antibacterial effects, such as disrupting the permeability of microbial cell membranes and cell walls, inhibiting protein synthesis, interfering with basic microbial processes, enhancing immune responses to pathogens, and causing disruption to their energy metabolism system.

## 3. Materials and Methods

### 3.1. Materials

The *H. marmoreus* powder was obtained from Shaanxi Snoot Biotechnology Co., Ltd. (Xi’an, China). The polyethersulfone separation ultrafiltration membrane with a relative molecular weight of 1, 5, and 10 kDa was obtained from Beijing Zhongke Ruiyang Membrane Technology Co., Ltd. (Bei’jing, China). The bovine serum protein (BSA) was obtained from Shanghai Aladdin Biochemical Technology Co., Ltd. (Shang’hai, China). Neutral protease (50,000 U/g), alkaline protease (200,000 U/g), composite protease (120,000 U/g), papain (800,000 U/g), and trypsin (250,000 U/g) were obtained from Shanghai Yuanye Biotechnology Co., Ltd. (Shang’hai, China).

### 3.2. Extraction of H. marmoreus Protein

*H. marmoreus* protein powder was mixed evenly with distilled water and then soaked for 30 min. The pH value of the mixture was adjusted using a 1 mol/L sodium hydroxide (NaOH) solution. The temperature for protein extraction is 50 °C, and the time is 30 min. During the extraction process, ultrasound-assisted treatment was used twice. When the solution temperature dropped to room temperature, the mixture was centrifuged at a speed of 7000 rpm for 25 min. The protein concentration in the supernatant was determined using the Coomassie Brilliant Blue staining method [38]. The average of the results of three parallel experiments was taken as the final measurement value.

Based on single-factor experiments, four factors significantly influencing the protein extraction rate were determined, including the solid–liquid ratio, pH value, ultrasonic power, and ultrasonic time period. Then, IBM SPSS Statistics 20 software (Armonk, NY, USA) was used to conduct orthogonal experiments with four factors and four levels to determine the optimal process for extracting *H. marmoreus* protein. The factors and levels of the orthogonal experiments are shown in Table 6.

### 3.3. Preparation of H. marmoreus Peptides

#### 3.3.1. Hydrolysis Process

Proteases were added to 50 mL of *H. marmoreus* protein solution and subjected to hydrolysis by heating and shaking at the optimal pH and temperature. Then, the mixture was heated in a 95 °C water bath for 15 min to inactivate the enzyme. Finally, after enzyme inactivation, the mixture was centrifuged at 6000× *g* rpm for 10 min. The obtained supernatant is the peptide solution [39].

#### 3.3.2. Screening of Proteases

The effectiveness of five proteases, including papain, neutral protease, trypsin, alkaline protease, and complex protease, in hydrolyzing *H. marmoreus* protein was analyzed. The hydrolysis conditions were as follows: substrate concentration of 4%, enzyme addition of 5000 U/g, and enzymatic hydrolysis time of 2 h. The temperature and pH were the optimal conditions for different enzymes. The optimal protease for enzymatic hydrolysis of *H. marmoreus* proteins was screened based on the hydrolysis degree.

#### 3.3.3. Single-Factor Experiment

The basic enzymatic conditions were set as a substrate concentration of 4%, temperature of 55 °C, pH value of 7, enzyme addition of 3000 U/g, and reaction time of 2 h. On this basis, different gradients were set for each parameter to examine the impact of different single factors on the enzymatic hydrolysis efficiency.

#### 3.3.4. Response Surface Test

According to the results of the single-factor experiments, the enzyme hydrolysis time (X_1_), substrate concentration (X_2_), pH value (X_3_), extraction temperature (X_4_), and enzyme dosage (X_5_) that had significant impacts on hydrolysis degree were selected as independent variables, and hydrolysis degree was selected as the response value for the Box–Behnken experiment. The five-factor and three-level experiments were designed and analyzed using Design Expert V8.0.6 software. The factor level codes are shown in Table 7.

#### 3.3.5. Determination of Hydrolysis Degree

Distilled water (25 mL) was added to protease hydrolysate (5 mL). The pH of the mixed solution was adjusted to 8.2 by NaOH standard solution. After adding 10 mL of neutral formaldehyde solution, the pH of the solution was adjusted to 9.2. The consumption volume of NaOH solution was recorded, and 30 mL distilled water was used as blank control. The formula for calculating the degree of hydrolysis (DH) was as follows:

$$X=\frac{\left(V_{1}-V_{2}\right)\times C\times 0.014}{V_{3}}$$

$$DH\left(\%\right)=\frac{X}{N}\times 100\%$$

where X was the content of amino nitrogen in the sample (g/100 mL); V_1_ was the volume (mL) of NaOH standard solution consumed after adding formaldehyde solution; V_2_ was the volume (mL) of NaOH standard solution consumed by the blank group after adding formaldehyde; V_3_ was the amount of sample taken (mL); C was the concentration of NaOH standard solution (mol/L); 0.014 was the milligram equivalent of nitrogen; N was the total nitrogen content of the sample (g/100 mL), measured by the Kjeldahl method.

### 3.4. Peptides’ Grading

Three different pore sizes (1, 5, 10 kDa) of ultrafiltration membranes were used to intercept peptides with different molecular weights. Four types of peptides were obtained: polypeptide-1 (<1 kDa), polypeptide-2 (1–5 kDa), polypeptide-3 (5–10 kDa), and polypeptide-4 (>10 kDa). The peptide contents were 0.239 mg/mL, 0.168 mg/mL, 0.152 mg/mL, and 0.135 mg/mL, respectively.

### 3.5. In Vitro Antioxidant Test

#### 3.5.1. Determination of DPPH Scavenging Ability

The DPPH scavenging ability of *H. marmoreus* peptides was determined using Karami’s method [40]. The DPPH scavenging rate was calculated based on the formula as follows:

$$Clearance\;rate(\%)=1-\frac{A_{0}-B}{A}\times 100\%$$

where *A*_0_ was the absorbance value measured by the sample; *B* was the absorbance value measured by replacing DPPH with anhydrous ethanol; *A* was the absorbance value of the DPPH solution in distilled water.

#### 3.5.2. Determination of •OH Scavenging Ability

The •OH scavenging ability of *H. marmoreus* peptides was determined using the method of Zhong [41]. The •OH scavenging rate was calculated based on the formula as follows:

$$•OHscavengingrate(\%)=1-\frac{A_{0}-B}{A}\times 100\%$$

where *A*_0_ was the absorbance value at 510 nm; *B* was the absorbance value using distilled water instead of salicylic acid at 510 nm; *A* was the absorbance value of distilled water at 510 nm instead of the sample.

#### 3.5.3. Determination of ·O_2_^−^ Scavenging Ability

The ·O_2_^−^ scavenging ability of *H. marmoreus* peptides was determined using the method of Esfandi et al. [42]. The ·O_2_^−^ scavenging rate was calculated based on the formula as follows:

$${\cdot O}_{2}^{-}scavenging\;rate(\%)=1-\frac{A-B}{A}\times 100\%$$

where *A* was the absorbance value of the self-oxidation rate of catechol using distilled water instead of a digestive solution; *B* was the absorbance value of the self-oxidation rate of catechol with an added digestive solution.

### 3.6. Determination of Antibacterial Activity

To evaluate the antibacterial activity of *H. marmoreus* peptides, we selected five common foodborne pathogenic micro-organisms, including four types of bacteria and a kind of fungus. *Aspergillus niger*, *Staphylococcus aureus*, *Salmonella typhimurium*, *Escherichia coli*, and *Pseudomonas aeruginosa* were common contaminating bacteria and conventional strains in food safety testing. To test the activity of *H. marmoreus* peptides in inhibiting harmful fungi, *Aspergillus niger* was selected as the tested fungus. It is a common food contamination fungus in daily life, which often leads to black mold in grains and fruits and vegetables, such as grapes [43], apricots [44], onions [45], peanuts [43], etc., causing food pollution or spoilage [43]. All micro-organisms were preserved in the National R & D Center for Edible Fungi Processing, Fuzhou, China.

Preparation of bacterial suspension: The tested bacteria were inoculated onto Beef Extract Peptone Agar medium and then cultured in a 37 °C incubator for 48 h to activate the strains. The activated bacterial strains were placed in 10 mL of sterile water and mixed well, and a bacterial suspension with a bacterial count of 1.45 × 10^8^ CFU/mL was prepared.

The bacterial counting method was as follows: The concentration of *Escherichia coli* solution cultured for 24 h was counted using a blood cell counting plate. The cell suspensions were diluted with sterile physiological saline to 1 × 10^8^, 2 × 10^8^, 4 × 10^8^, 6 × 10^8^, 8 × 10^8^, 1 × 10^9^, and 1.2 × 10^9^ bacterial counts per milliliter. After shaking the bacterial solution evenly, its optical density (OD) value was measured in a 1 cm colorimetric dish at a wavelength of 560 nm. The standard curve was plotted with the OD value as the vertical axis and the number of cells per milliliter as the horizontal axis. The microbial solution to be tested was appropriately diluted with sterile physiological saline and shaken evenly, and its optical density was measured using the same method. According to the measured OD value, the bacterial count per milliliter (CFU/mL) could be obtained from the standard curve.

Preparation of fungal suspension: *Aspergillus niger* was inoculated onto Potato Dextrose Agar medium and incubated at 26 °C for 72 h to activate the strain. The surface of *A. niger* culture medium was repeatedly washed with 1 mL sterile physiological saline, causing its mycelium to fall off. Then, the bacterial suspension was carefully transferred and diluted with sterile physiological saline to the same OD value as the bacteria.

The bacterial suspension was evenly coated on plates containing the corresponding culture medium, and each plate was coated 3 times, with 1/3 of each time. After uniform coating, three sterilized Oxford cups were evenly and equidistantly placed on a flat plate, arranged in an equilateral triangle. A volume of 0.2 mL of 4 mg/mL peptide solution sample was added to an Oxford cup and diffused at 4 °C for 4 h. Bacteria were cultured at 37 °C for 24 h, while the fungi were cultured at 26 °C for 48 h. The growth of colonies was observed, and the diameter of the inhibition zone was measured using the cross method.

### 3.7. Statistical Analysis

The test data were analyzed by variance using the statistical analysis software SPSS 20. Multiple comparisons were performed using Duncan’s method, and the significance level was set as *p* < 0.05. Response surface analysis of the data was carried out using Design-Expert13 software. All graphs were created using the Origin2022 software for plotting. All data were averaged by repeating the results of three experiments.

## 4. Conclusions

In the present work, the ultrasound-assisted extraction method was used to extract protein from *H. marmoreus*. The process parameters, including solid–liquid ratio, pH value, ultrasonic power, and ultrasonic time, were optimized. The effectiveness of five enzymes in hydrolyzing seafood mushroom protein was analyzed, and trypsin, with the highest hydrolysis rate, was selected as the optimal enzyme for preparing seafood mushroom peptides. The hydrolysis process was studied using single-factor and response surface experiments, and the optimal hydrolysis process was determined through regression model analysis. Four types of seafood mushroom peptides were prepared by molecular weight grading, which showed significant scavenging effects on DPPH•, •OH, and ·O_2_**^−^** free radicals, as well as strong antibacterial activity. Among them, low-molecular-weight peptides had stronger antioxidant [46] and antibacterial activities [47]. This study provides a theoretical basis for the efficient preparation of seafood mushroom peptides and the development of antioxidant and antibacterial peptide products. As a natural, healthy, and highly bioactive substance, the application of edible mushroom peptide is not only limited to the food field but will be more widely covered in the future in medicine [48] and skin care [49]. It is believed that the bioactive peptide of edible mushrooms will attract more researchers from all walks of life to explore it under the broad application prospect.

## Figures and Tables

**Table 1:** Experimental results and range analysis of *H. marmoreus* protein extraction process.

Test Number	Solid–Liquid Ratio (A)	pH (B)	Ultrasonic Power (C)	Ultrasonic Time (D)	Empty Column (E)	Percent Extraction Rate (%)
1	1	1	1	1	1	37.2
2	1	2	2	2	2	39.6
3	1	3	3	3	3	47.8
4	1	4	4	4	4	41.3
5	2	1	2	3	4	49.8
6	2	2	1	4	3	46.6
7	2	3	4	1	2	42.1
8	2	4	3	2	1	46.9
9	3	1	3	4	2	48.3
10	3	2	4	3	1	52.6
11	3	3	1	2	4	45.3
12	3	4	2	1	3	41.7
13	4	1	4	2	3	37.5
14	4	2	3	1	4	38.5
15	4	3	2	4	1	39.2
16	4	4	1	3	2	48.9
K1	165.9	172.8	178	159.5	175.9	
K2	185.4	177.3	170.3	169.3	178.9	
K3	187.9	174.4	181.5	199.1	173.6	
K4	164.1	178.8	173.5	175.4	174.9	
k1	41.48	43.20	44.50	39.88	43.98	
k2	46.35	44.33	42.58	42.33	44.73	
k3	46.98	43.60	45.38	49.78	43.73	
k4	41.03	44.70	43.38	43.85	43.73	
R	5.95	1.5	2.8	9.9	1	
Excellent level	A3	B4	C3	D3		

**Table 2:** Analysis of variance for extraction process.

Source of Differences	SS	df	MS	F	Significance
A	118.3669	3	39.4556	31.0115	**
B	5.5519	3	1.8506	1.4546	
C	18.2169	3	6.0723	4.7727	
D	212.7469	3	70.9156	55.7385	**
E	3.8169	3	1.2723		
Total	358.6994	15			

F_0.05_(3,3) = 9.2766, F_0.01_(3,3) = 29.4567; ** Extremely significant.

**Table 3:** Response surface test plan and results.

Number	Enzymatic Hydrolysis Time X_1_ (h)	Substrate Concentration X_2_ (%)	pH Value X_3_	Extraction Temperature X_4_ (°C)	Enzyme Dosage X_5_ (U/g)	Hydrolysis Degree (%)
1	−1	−1	0	0	0	47.19
2	1	−1	0	0	0	51.59
3	−1	1	0	0	0	38.06
4	1	1	0	0	0	39.38
5	0	0	−1	−1	0	48.12
6	0	0	1	−1	0	49.06
7	0	0	−1	1	0	46.31
8	0	0	1	1	0	48.01
9	0	−1	0	0	−1	43.61
10	0	1	0	0	−1	36.13
11	0	−1	0	0	1	52.03
12	0	1	0	0	1	41.91
13	−1	0	−1	0	0	44.62
14	1	0	−1	0	0	50.71
15	−1	0	1	0	0	45.73
16	1	0	1	0	0	52.69
17	0	0	0	−1	−1	44.88
18	0	0	0	1	−1	43.78
19	0	0	0	−1	1	52.41
20	0	0	0	1	1	51.97
21	0	−1	−1	0	0	47.93
22	0	1	−1	0	0	36.85
23	0	−1	1	0	0	48.62
24	0	1	1	0	0	38.28
25	−1	0	0	−1	0	46.97
26	1	0	0	−1	0	53.53
27	−1	0	0	1	0	47.41
28	1	0	0	1	0	54.06
29	0	0	−1	0	−1	43.56
30	0	0	1	0	−1	46.03
31	0	0	−1	0	1	54.34
32	0	0	1	0	1	53.29
33	−1	0	0	0	−1	49.06
34	1	0	0	0	−1	48.45
35	−1	0	0	0	1	50.65
36	1	0	0	0	1	56.76
37	0	−1	0	−1	0	47.35
38	0	1	0	−1	0	39.82
39	0	−1	0	1	0	47.35
40	0	1	0	1	0	39.71
41	0	0	0	0	0	56.81
42	0	0	0	0	0	55.88
43	0	0	0	0	0	58.13
44	0	0	0	0	0	56.81
45	0	0	0	0	0	56.21
46	0	0	0	0	0	56.59

**Table 4:** Analysis of variance of regression model equations.

Source of Variation	Sum of Squares	Degree of Freedom	Mean Square	F-Value	*p*-Value	Significance
X_1_	87.80	1	87.80	44.14	<0.0001	**
X_2_	356.55	1	356.55	179.26	<0.0001	**
X_3_	5.37	1	5.37	2.70	0.1129	
X_4_	0.78	1	0.78	0.39	0.536	
X_5_	209.24	1	209.24	105.20	<0.0001	**
X_1_X_2_	2.37	1	2.37	1.19	0.2853	
X_1_X_3_	0.19	1	0.19	0.095	0.7603	
X_1_X_4_	0.00203	1	0.00203	0.00102	0.9748	
X_1_X_5_	11.29	1	11.29	5.68	0.0251	*
X_2_X_3_	0.14	1	0.14	0.07	0.7952	
X_2_X_4_	0.00303	1	0.00303	0.00152	0.9692	
X_2_X_5_	1.74	1	1.74	0.88	0.3583	
X_3_X_4_	0.14	1	0.14	0.073	0.7898	
X_3_X_5_	3.10	1	3.10	1.56	0.2236	
X_4_X_5_	0.11	1	0.11	0.055	0.8169	
X_12_	66.49	1	66.49	33.43	<0.0001	**
X_22_	790.02	1	790.02	397.19	<0.0001	**
X_32_	188.93	1	188.93	94.98	<0.0001	**
X_42_	146.96	1	146.96	73.88	<0.0001	**
X_52_	102.49	1	102.49	51.53	<0.0001	**
Model	1523.01	20	76.15	38.29	<0.0001	**
Residual	49.73	25	1.99			
Misfit term	46.74	20	2.34	3.91	0.068	
Pure error	2.98	5	0.60			
Total variation	1572.73	45				

* Different at the 0.05 level; ** Significantly different at the 0.01 level.

**Table 5:** Antibacterial effects of *H. marmoreus* peptides (antibacterial diameter: mm).

Sample	*Staphylococcus aureus*	*Salmonella typhimurium*	*Escherichia coli*	*Pseudomonas aeruginosa*	*Aspergillus niger*
Polypeptides-1	11.37 ± 0.48 ^a^	12.03 ± 0.22 ^a^	13.51 ± 0.42 ^a^	12.72 ± 0.68 ^a^	7.13 ± 0.25 ^b^
Polypeptides-2	7.08 ± 0.35 ^b^	4.62 ± 0.16 ^d^	0.00 ± 0.00 ^c^	9.17 ± 0.43 ^c^	6.98 ± 0.36 ^b^
Polypeptides-3	10.11 ± 0.67 ^a^	9.71 ± 0.56 ^b^	6.77 ± 0.39 ^b^	10.58 ± 0.39 ^ab^	0.00 ± 0.00 ^c^
Polypeptides-4	0.00 ± 0.00 ^c^	7.92 ± 0.28 ^c^	5.99 ± 0.32 ^b^	8.76 ± 0.19 ^c^	8.95 ± 0.32 ^a^

Different letters in the same indicators indicate significant differences (*p* < 0.05).

**Table 6:** Factors and levels of orthogonal experiments for *H. marmoreus* protein extraction.

No.	Solid–Liquid Ratio (g:mL)	pH	Ultrasonic Power (W)	Ultrasonic Time (min)
1	1:25	10	200	25
2	1:30	11	250	30
3	1:35	12	300	35
4	1:40	13	350	40

**Table 7:** Experimental factors and levels for response surface test.

No.	Enzymatic Hydrolysis Time X_1_ (h)	Substrate Concentration X_2_ (%)	pH Value X_3_	Extraction Temperature X_4_ (°C)	Enzyme Dosage X_5_ (U/g)
−1	2.5	3	6	50	4000
0	3.0	4	7	55	5000
+1	3.5	5	8	60	6000

## Data Availability

Data are contained within the article.
